# The Effect of Plating, Ingredients, and Cooking Processes on the Acceptance and Authenticity of Ethnic Rice Dishes

**DOI:** 10.3390/foods9080976

**Published:** 2020-07-23

**Authors:** Cho-Long Lee, Soo-Hyun Lee, Ga-Gyeong Seo, Jae-Hee Hong

**Affiliations:** 1 Department of Food and Nutrition, Seoul University, Seoul 03080, Korea; cholone2@snu.ac.kr (C.-L.L.); tjrkrud94@snu.ac.kr (G.-G.S.); 2 Research Institute of Human Ecology, Seoul University, Seoul 03080, Korea; jinlee0123@gmail.com

**Keywords:** ethnic food, fusion food, liking, expectation, familiarity, ethnic authenticity, localization, cross-cultural study

## Abstract

Familiarity and ethnic authenticity have a significant influence on the liking of ethnic food. Thus, it is crucial to identify the degree to which a dish can be modified in order to increase hedonic responses and familiarity without the loss of ethnic authenticity. This study determined the degree to which perceptions of the Korean rice dish, bibimbap, would vary upon modification of its ingredients, cooking process, or plating using the Southeast Asian market as a model system. The dish was prepared in Korean style or as Nasi Goreng, the Southeast Asian style. Eight formulations (2 ingredients × 2 cooking methods × 2 plating styles) were tested by panels, including 77 Southeast Asians and 72 Koreans. Hedonic responses, familiarity, ethnic authenticity, and purchase intent were evaluated using a nested analysis of variance. Ingredients and cooking methods had a significant influence on liking and perceived ethnic authenticity. In addition, plating had a substantial effect on the perception of ethnic authenticity and expected liking. Overall, the rate of positive responses increased when region-specific cooking processes and plating were matched. Taken together, our results suggest that modification of familiar dishes needs to be carefully considered as it can have complex effects on liking and perceived ethnic authenticity.

## 1. Introduction

The growth of international trade, the population migration, and tourism have all contributed to the increasing demand for ethnic foods. In 2018, the global ethnic food market was valued at 36,481.1 million USD; forecasts suggest a compound annual growth rate of 11.8% during 2019–2024 [1]. As part of this trend, many countries have promoted the export of ethnic foods; it enhances the sustainability of local agriculture, promotes tourism, and improves the national image [2]. As multiculturalism has emerged as a societal trend worldwide [3], consumers have expressed a great interest in sampling foreign or ethnic foods. As such, there are fewer barriers to introducing foods with unusual tastes and flavors.

Southeast Asian countries have emerged as a potential market for Korean foods. There is a growing interest in Korean foods among young Southeast Asians owing to the influence of the Korean Wave [4]. This trend is particularly noticeable in Malaysia and Indonesia, which are regions considered to be the leading Southeast Asian countries due in part to their large population (300 million) and rapid economic growth. The Malaysian food market is expanding at an average rate of 7.9% per year. The processed food and catering industries in Indonesia have also grown at an annual rate of 10.6% during the past 5 years [5]. In these countries, Korean food has been introduced as one of the cultural contents and has recently been the subject of significant interest and high recognition [6]. Recent reports indicate that 67.7% of Malaysian and Indonesian consumers consume Korean food, and 47.3% had dined at Korean restaurants in their home countries; moreover, 20.4% of Southeast Asian consumers reported that they cooked and ate Korean food [7]. Therefore, the Southeast Asian food market, including Malaysia and Indonesia, has become a major target for the export of Korean ethnic food.

Until recently, explorations of the cross-cultural appreciation of ethnic foods focused on certain concepts, such as familiarity and fear of new foods and flavors (neophobia). Familiarity with specific food products has a clear effect on food-associated beliefs, potential acceptability [8], and food choices [9]; research suggested that familiarity provided a context for new foods and assurance of their pleasing taste and safety. Lack of familiarity results in negative responses, including risk and suspicion; this will have an undesirable effect on hedonic responses, openness to new foods, and purchase intent [10]. Familiarity is achieved through previous experience and exposure; likewise, repeated exposure has a positive effect on one’s appreciation for a new food by reducing uncertainty about safety and identity [11]. Several studies have shown that consumers exhibited more favorable responses to ethnic foods once they had been familiarized through previous exposures or to ethnic foods that had sensory characteristics similar to foods from their home countries [12,13,14].

The effect of familiarity on liking for ethnic foods may have a practical implication that it can be considered as one strategy to improve liking for an ethnic food. For example, familiarity of an ethnic food may be increased by applying a target country’s “flavor principles”, which are unique flavor combinations serving to distinguish the cuisine of one country from those of other regions and nationalities [15]. However, when the flavor of bulgogi, traditional Korean barbecued beef, was adjusted in order to appeal to American consumers’ tastes, the modified sample was not significantly preferred over the original Korean recipe. This finding suggests that flavor modification does not necessarily improve consumer liking of ethnic foods [16]. Furthermore, extreme modification using the flavor principle may fail to satisfy consumer’s desire to experience new and exotic flavors [17], which remains one of the main reasons for consuming ethnic foods [17,18]. Therefore, it is necessary to understand how the balance of familiarity and food authenticity perceived by consumers affects the liking for and perception of ethnic foods.

An exotic sensory experience representing the unique characteristics of a foreign culture, a concept known as ethnic authenticity, is among the key drivers of ethnic food consumption [17]. With regard to ethnic foods, terms such as “traditional”, “authentic”, “ethnic”, “exotic”, “unique”, and “specialty” have been used interchangeably in the previous studies to present the concept of ethnic authenticity. A more carefully defined concept of ethnic authenticity was recently suggested [19]. Authenticity focuses on issues relating to ingredients and dishes unique to a geographical place; likewise, the concept of authenticity extends to a consideration of whether the food is cooked in a traditional way, prepared with local ingredients and by natives [19]. Previous studies have identified factors contributing to ethnic authenticity, including the ingredients, cooking processes (methods or style), place of origin, taste, and flavors [20,21,22]. Among these factors, ingredients and cooking processes are identified as among the most common factors contributing to ethnic authenticity.

Ingredients that are locally sourced or unique are considered to be among the most important elements contributing to the perceptions of ethnic authenticity [23]. There is an additional support for this notion, [24] reported that the use of authentic Thai ingredients was the most critical factor contributing to an authentic experience. Additionally, Chinese food is distinguished from other ethnic foods by its use of several local ingredients [20]. We expected that the use of different and unfamiliar ingredients would have a significant effect on consumer liking [25]. Moreover, preparation and cooking styles can also be a marker of authenticity [26]. Molz pointed out that traditional ingredients and culinary techniques are markers expressing authenticity [27]. Other studies also suggested that the cooking process or style might be used as a tool for judging the authenticity of an ethnic dish [20,21,22].

In addition to these factors, plating may influence the perception of ethnic authenticity. Even if the same ingredients and cooking processes were applied, different plating styles can elicit a completely different impression of the ethnic food product. Recent studies reported that visual cues, such as dishware and plating, could divert attention from the flavors of the dish [28]. Subtle changes in the plating influenced the perception of flavor and liking [29]. For example, when meal ingredients were presented in a neat and orderly manner, subjects rated the taste more positively than when ingredients were presented in a disorderly fashion [30]. Additionally, changes in organization and visual arrangement of food elements increased overall liking and willingness to purchase [31]. Obviously, what consumers see on the plate rapidly generates expectations and liking about sensory attributes, including taste and flavor [32]. In addition, in a cross-cultural study of plating, there was a common preference with respect to the number of different colors and components and also the fill level of a plate. However, there were diverging cross-cultural preferences with respect to the position of the main component of the dish, organization of components, and casual vs. a formal presentation [29].

Based on the results from the aforementioned studies, we hypothesize that the ingredients, cooking process, and plating style all have an effect on the liking, perceptions of familiarity and ethnic authenticity, and purchase intent for ethnic rice dishes. This hypothesis has been mainly studied using surveys [17,33] or scenarios [34,35], but has not been examined using a controlled experimental design in a model food system. Therefore, the present study was conducted to explore how modification of three major components (ingredients, cooking process, and plating) affect consumer liking, perceived familiarity, ethnic authenticity, and purchase intent. The hypothesis of the present study was examined in a simulated real-life situation, in which bibimbap, a popular Korean rice dish, was localized and provided to consumers in Southeast Asian countries.

## 2. Materials and Methods

### 2.1. Sample

The formulations were designed assuming a real-life situation, in which a prototype of the Korean rice dish, bibimbap (Korean mixed rice with meat and assorted vegetables), was modified by incorporating Southeastern culinary styles. Bibimbap was selected as a base food system, considering that it is quite popular and has been consumed by 41.6% of Southeastern Asian consumers who report experience with Korean foods [6]. Furthermore, Southeast Asian consumers are generally familiar with rice dishes, as rice is a staple food in this region.

The three main components of the rice dishes, i.e., ingredients, cooking process, and plating, were adjusted to either Korean (K) style or Southeast Asian (SA) style, respectively. Nasi Goreng (Indonesian fried rice), the popular and widely consumed Southeast Asian rice dish, was used as a reference for SA-styled components. A total of eight different combinations of three components in two different ethnic styles (K and SA) were prepared (Table 1). The cooking procedures and ingredients are presented in Appendix A. Through the literature review and interviews with Korean, Malaysian, and Indonesian consumers, we determined that the key components for K plating included a black porcelain bowl and sprinkled sesame seeds; contrarily, SA plating was signified with a banana leaf and cucumber slices as garnish. Malaysians and Indonesians who reside in Korea provided input on whether the SA rice dish was presented in a typical SA-style. 

The dishes to be evaluated for their appearance were prepared separately from those to be evaluated for flavor and texture. This set of dishes was presented as an entire portion prepared in a K or SA plating style. For tasting, 30 ± 1 g of each sample was provided in a white disposable plastic bowl (70 mL). The samples were presented immediately after cooking, as requested by the panelists. The temperature of the samples at the time of tasting was 60 ± 1 °C. Warm water (45 ± 1 °C) was provided for mouth rinsing. The samples for appearance evaluation and for tasting were coded with unique three-digit random numbers.

### 2.2. Subjects

Our main aim in this study was to determine the responses of SA consumers to ethnic foods that had been modified by incorporating components from their culinary culture; we were also interested in identifying elements of a cross-cultural comparison between SA and Korean consumers. Among SA subjects that have diverse culinary backgrounds, the community whose diet is based on Malay cuisine was considered. Therefore, the SA panel was comprised of Malaysian and Indonesian consumers. These participants share not only the same dietary culture but also the same history, geographical proximity, cultural heritage, and religion [36]; as such, they can be considered as a relatively homogeneous cultural community compared with other SA countries. A similar approach was taken in a previous study [37] that compared sensory perception and verbal expression among Koreans, Chinese, and Westerners who were recruited based on the shared language and similar food culture.

The Southeast Asian subjects (SA panel) included 52 Malaysians and 25 Indonesians who were residing in Korea; the participants were recruited by posting online flyers in the Malaysian and Indonesian communities using convenience and a snowball sampling scheme. Korean participants (*n* = 72) were also recruited by posting flyers, both on campus and online. Individuals who were allergic to the ingredients and those who were or who might be pregnant were excluded from this study. The research protocol was approved by the Institutional Review Board at Seoul National University (IRB No: 1802/003-002).

### 2.3. Test Procedure

Before the test, a brief introduction to the procedure was provided to the panelists. An entire one-serving-sized sample was presented to the panelists for the evaluation of its appearance. After evaluating the appearance, the panelists then tasted a smaller portion of each sample (30 ± 1 g) and evaluated overall liking, flavor and texture liking, perceived ethnic authenticity and familiarity, and purchase intent. Samples were presented following a complete randomized block design. The panelists took a 1-min break and rinsed their mouths with warm water between samples. To decrease physical and mental fatigue, panelists were asked to take a mandatory 5-min break after tasting the first four samples. Perceived Korean ethnic authenticity (K ethnic authenticity) and Southeast Asian ethnic authenticity (SA ethnic authenticity) were evaluated by a rating system that asked the participants to score their perceptions as to how much the sample appeared to be K- or SA-style using a 7-point category scale (1 = not at all, 7 = very much) [38]. Familiarity was rated on a 9-point category scale (1 = not familiar at all, 9 = extremely familiar) [33]. Purchase intent was rated on a 7-point Likert scale (1 = strongly disagree, 7 = strongly agree) [39,40]. Liking were rated on a 9-point category scale (1 = dislike very much, 9 = like very much). After completing the tasting test, the panelists filled out a questionnaire focused on demographic information, previous experiences with Korean or SA foods, food neophobia (FN) score [41], and variety seeking (VS) score [42]. The test was conducted in individual booths and took approximately 1 h.

### 2.4. Statistical Analysis

A three-way analysis of variance (ANOVA) was conducted to evaluate the effect of independent variables (plating, ingredient, cooking process, and nationality of panel participants) on liking, familiarity, perceived Korean or SA, and purchase intent. The panel was nested with respect to nationality. The ANOVA model included the effect of the main factors and their secondary and tertiary interactions. Duncan’s multiple range test or *t*-test was conducted as a post hoc analysis. In addition, correlation analysis was conducted to determine the relationships between the dependent variables. The results of demographic information and previous food experiences were analyzed by counting the frequency of each response. Individual scores of FN and VS were obtained as the sum of the ratings given to the statements comprising each scale. The ratings of the statements suggesting neophobia or VS were reversely recorded prior to analysis. FN and VS scores were compared between the two panels using independent *t*-test. The SPSS statistical software package (ver. 23, IBM, New York, NY, USA) was used, and the level of significance was set at *p* < 0.05.

## 3. Results

### 3.1. Characteristics of Korean and Southeast Asian Panels

The findings in Table 2 revealed that most of the members of the SA panel were female (70.1%), Malaysians (67.5%), and 20–29 years old (79.2%). Most (79%) of the members of the SA panel had resided in Korea for more than 1 year, and 32.5% had been in Korea for more than 3 years. Moreover, 83.1% of the members of the SA panel had consumed Korean food in the past for a period of more than 1 year, whereas 42.9% consumed Korean food every day. Most of the participants had sampled bibimbap before the trial, and more than half (51.9%) of the SA panel reported eating bibimbap two or more times per month. Similarly, the K panel was comprised of 46% of men and 54% of women. Most of the members of the K panel (93.1%) have consumed SA foods in the past, primarily Vietnamese (83.3%) and Thai (62.5%) cuisines. After tasting the samples provided, 68.2% of the members of the K panel reported that they had tasted a Southeastern rice dish that was similar to the samples; this result suggested that they had familiarity with the SA-style samples. Overall, the K panel had a significantly higher FN score (54.7 ± 10.6) than the SA panel (51.0 ± 10.2), indicating more neophobic tendencies (*p* < 0.05). VS scores did not differ significantly between the two panel groups.

### 3.2. Effect of the Main Factors and Their Interactions with Liking, Perceived Ethnicity, Familiarity, and Purchase Intent

#### 3.2.1. Main Effect

The nationalities of each member of the panel had a significant influence on the factors evaluated, including hedonic ratings, perceived ethnic authenticity, and purchase intent, but not familiarity (Table 3). The SA panel rated higher than the K panel for variables associated with liking and purchase intent, but not flavor liking (Table 4); this result indicated that the members of the SA panel were less likely to reject modified rice dish samples. Conversely, the members of the K panel appeared to expect the specific characteristics of bibimbap that fits to its traditional image; these may have been formed more clearly in response to previous experience and knowledge [43]. As such, the members of the K panel might be more reluctant to accept modified versions of bibimbap.

The use of K ingredients resulted in significantly higher ratings with respect to all hedonic attributes, familiarity, and purchase intent compared with ratings in response to SA ingredients (Table 3 and Table 4). The nature of the ingredients had a particularly significant effect on perceived ethnic authenticity; this result suggests that cultural and national identification with specific ingredients is a major component of the perception of ethnic authenticity. Furthermore, we confirmed that the ingredients associated with different nationalities included in this study had a significant influence on the recognition of cuisine ethnicity. The results in Table 4 indicate that perceived ethnic authenticity was highly dependent on the use of the ingredients from the corresponding country.

The cooking process also significantly influenced all hedonic ratings, perception of ethnic authenticity, and purchase intent (Table 3). K and SA cooking processes affected hedonic ratings in different ways. The K cooking process was associated with a significantly increased mean positive appearance score compared with the SA cooking process (Table 4). Similarly, the SA cooking process significantly increased the liking scores after tasting, the purchase intent, and the perception of SA ethnicity compared with variables associated with the K cooking process.

Plating significantly influenced only the appearance liking and perceived K ethnic authenticity (Table 3). Appearance was significantly preferred when K plating was used compared with SA plating (Table 4). Additionally, although not significant, perceived K ethnicity tended to be higher when K plating was used rather than SA plating.

#### 3.2.2. Two-Way Interactions

**Figure 1:**
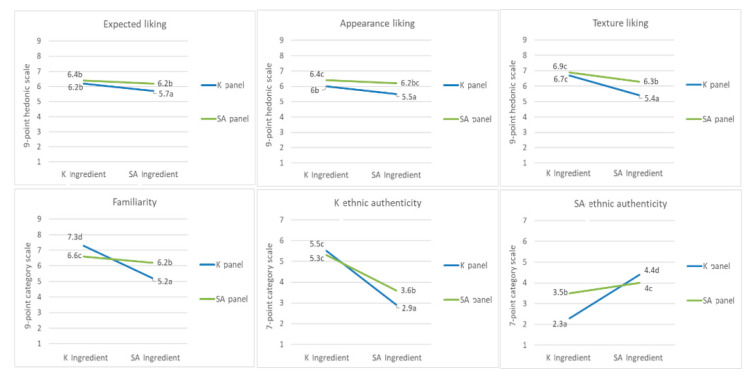
Effect of the nationality of the panel × ingredient on the expected liking, appearance liking, texture liking, familiarity, and K and SA ethnic authenticity, different letters indicate significant difference.

The significant interactions between the nationalities of the members of the panel and each of the three main factors (ingredients, cooking process, and plating) were observed (Table 3). The interaction between the nationalities of the members of the panel and ingredients had a significant effect on the expected liking, appearance and texture liking, familiarity, and perceived K and SA ethnic authenticity (Figure 1). When SA ingredients were used in the sample, the K panel exhibited a more significant decrease in liking for expected liking, appearance, and texture liking compared with the SA panel. The interaction effect of the nationality and ingredients of the panel on the familiarity was quite apparent. In particular, when SA ingredients were used, the members of the K panel reported a substantial decrease in familiarity. Interestingly, the SA panel rated the samples with K ingredients as significantly more familiar than those with SA ingredients. This may be attributed to the fact that most of the SA panelists recruited for this study had resided in Korea for more than 1 year and had therefore become familiar with Korean ingredients. When K ingredient was used in place of the SA ingredient, both panels’ perception of K ethnic authenticity decreased and perceived SA ethnic authenticity increased. However, when SA ingredient was used, K panel’s ratings of K ethnic authenticity fell to a greater extent, and that of SA ethnic authenticity rose to a greater extent compared to SA panel’s ratings.

**Figure 2:**
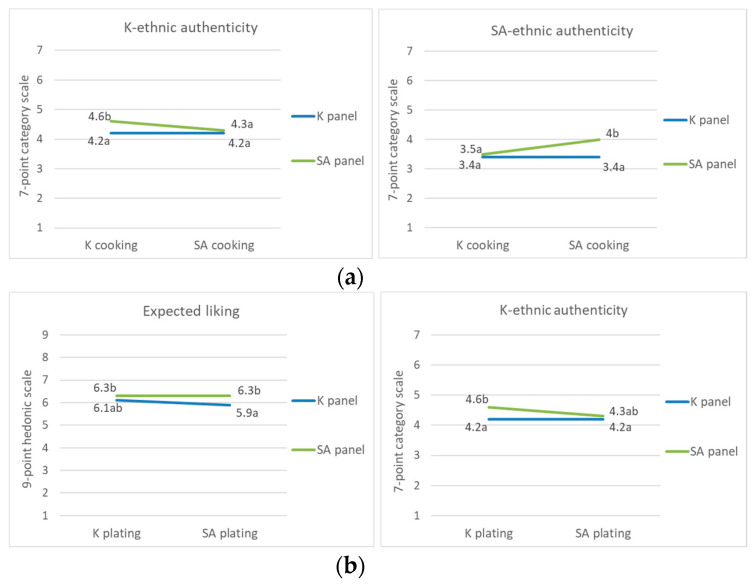
Effect of the (**a**) nationality × cooking process on K and SA ethnic authenticity and (**b**) nationality × plating on the expected liking and K ethnic authenticity, different letters indicate significant difference.

The interaction between nationalities and the cooking process significantly influenced only the perception of ethnic authenticity. Different cooking processes did not influence the K panel members’ perception of ethnic authenticity (Figure 2a). Contrarily, the SA panel perceived ethnic authenticity that was consistent with the nationality associated with the cooking process.

Plating interacted with the panel members’ nationalities and this interaction significantly influenced expected liking and K ethnic authenticity (Table 3). Although the difference was not significant, the SA panel’s expected liking was not affected by the style of plating, while the K panel’s expected liking was slightly decreased in response to SA-style plating (Figure 2b). In contrast, plating had no effect on the K panel’s perception of K ethnic authenticity, although the use of SA plating slightly decreased the SA panel’s perception of K ethnic authenticity.

**Figure 3:**
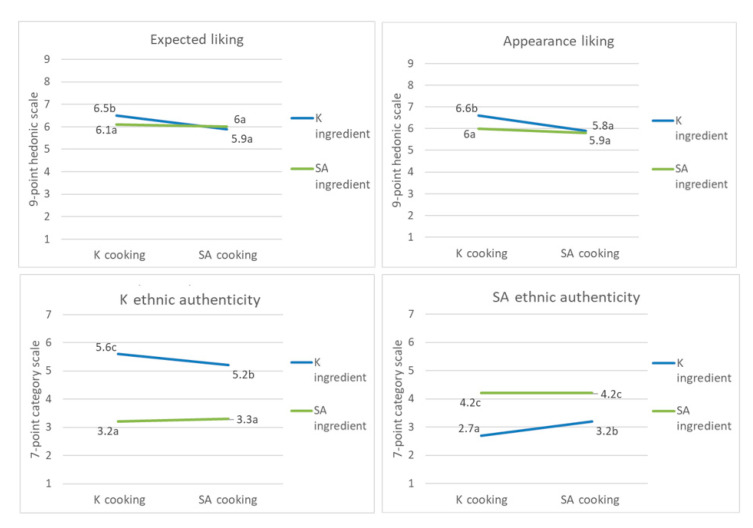
Effect of the ingredient × cooking process on expected liking, appearance liking, and K and SA ethnic authenticity, different letters indicate significant difference.

The interactions between ingredients and the cooking process had a significant effect on the expected liking, appearance liking, and perceived ethnic authenticity (Table 3). Expected liking and appearance liking were significantly higher when the K cooking process (mixing) was applied using K ingredients (Figure 3). However, the expected liking and appearance liking of the samples using SA ingredients were not affected by the cooking process. Moreover, when the SA cooking process (stir-frying) was applied to K ingredients, the perception of K ethnic authenticity was significantly decreased and that of SA ethnic authenticity was significantly increased.

#### 3.2.3. Three-Way Interactions

**Figure 4:**
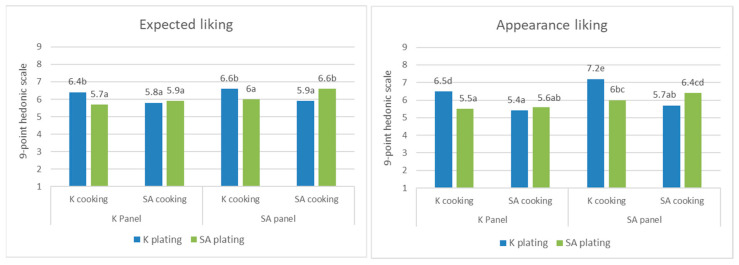
Effect of the nationality × cooking process × plating on expected liking and appearance liking, different letters indicate significant difference.

Three-way interactions among the nationalities of the panel, cooking process, and plating significantly affected the expected liking and appearance liking (Table 3). The two panel groups highly rated expected liking and appearance liking for samples when the cooking process and plating style originated from the same country (Figure 4). The members of the K panel rated the expected and appearance liking highly only for the samples that used the K cooking process and K plating. Conversely, the SA panel highly evaluated the expected liking and appearance likings when the nationality of the cooking process and plating of the samples was the same. However, the effect of the three-way interaction was limited to the evaluation of appearance only, excluding the evaluation of flavor and texture.

### 3.3. Correlation between Dependent Variables

Table 5 presents the correlation coefficients among the outcome variables for each panel group. There were significant correlations among perceived ethnicity, familiarity, overall liking, and purchase intent. The K panel exhibited a high positive correlation between K ethnic authenticity and familiarity. Moreover, the K panel members’ perceived familiarity positively correlated with the overall liking and purchase intent. However, SA ethnic authenticity exhibited a high negative correlation with familiarity. Likewise, the perception of SA ethnic authenticity correlated negatively with the overall liking and purchase intent. Contrarily, in the case of the SA panel, there was a positive correlation between SA ethnic authenticity and familiarity, but this was weaker than the correlation between K ethnic authenticity and familiarity observed for the K panel. The findings from the SA panel also included a weak but significant positive correlation between K ethnic authenticity and familiarity, as well as between K ethnic authenticity and overall liking.

## 4. Discussion

### 4.1. Effect of Adjustment of Components in an Ethnic Rice Dish

All components had a significant effect on response variables (Table 3). However, among them, the factor ingredients exhibited the largest F-values for all responses, except expected liking and appearance liking; this result indicated that the nature of ingredients used to prepare the rice dish explained the largest proportion of the total variance for almost all measures. Moreover, two-way interactions including ingredients revealed a significant effect on the expected liking, appearance liking, and ethnic authenticity. This suggests that the ingredients used to prepare the ethnic rice dish are the major determinants of liking and perceived ethnic authenticity.

The cooking process was a variable that had an effect on the overall hedonic rating and perceived ethnic authenticity. In particular, the SA panel’s perceived ethnic authenticity varied according to the nationality of the cooking process (Figure 2a). This result is consistent with those of previous studies that have reported that intrinsic cooking processes symbolize ethnic authenticity [20,21,22,26].

**Figure 5:**
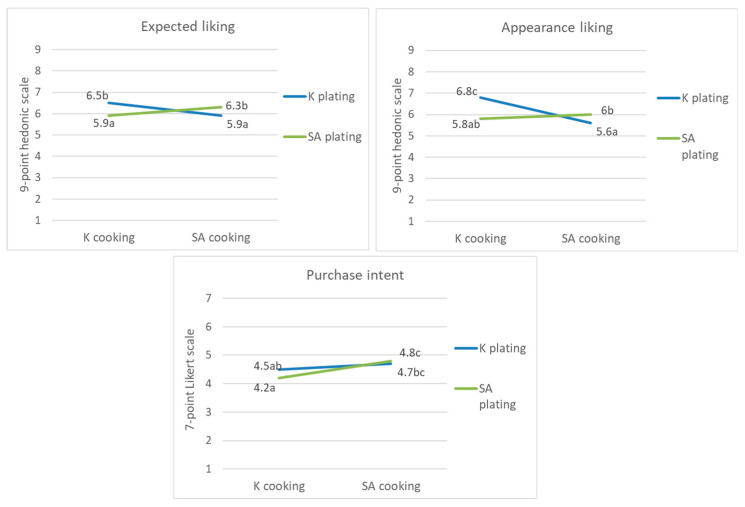
Effect of the cooking process × plating on expected liking, appearance liking, and purchase intent, different letters indicate significant difference.

Plating had an effect on the expected liking, appearance liking, and purchase intent via interactions with the cooking process (Table 3). In particular, the cooking process and plating appear to be closely related to one another, as all panels rated liking scores significantly higher when this two component’s ethnicity were matched (Figure 4 and Figure 5). Ratings of purchase intent show a slightly different pattern from that of liking scores. Purchase intent was rated higher when SA cooking was used than when K cooking was used, but the degree of increase appeared greater when both cooking process and plating followed SA-style than when they followed K-style. The most immediate explanation for this result is that plating should be organized with respect to the way in which the ingredients were cooked or processed [44]. In this study, the panelists may have perceived that the plating did not appropriately reflect the cooking method when inconsistencies existed between the cooking process and plating. Processing visual cues from the sample is influenced by the cooking process, which is an incidental aspect other than plating [45]. This might have had a negative effect on both the expected liking and appearance liking. In addition, the panel’s previous food experiences might have evoked a sense of incongruence when the nationalities of the plating and cooking process were not matched.

In general, interactions between components revealed that maintaining consistency of ethnic styles increased both the liking and perception of ethnic authenticity. According to cognitive dissonance theory [46], when belief, values, perceptions, behaviors, and attitudes are not consistent with one another, a negative psychological status known as dissonance occurs, which are associated with negative responses to stimuli. We hypothesized that incongruent ethnic styles of the components result in cognitive dissonance, thereby promoting negative responses to the food samples. Previous studies [38,47] reported that ethnic congruence of food and environmental cues, including music, décor, and food names, all have an effect on the perception of ethnicity and food choice; these factors lead to the selection of foods that were ethnically congruent with the environmental cues. However, the environmental cues hardly affected liking for the food [38]. Although more research into this topic is required, it can be suggested that extrinsic cues hardly affected liking, whereas intrinsic cues, as observed in this study, had more of a critical effect on liking for foods. This argument should be examined in the future studies.

### 4.2. Effects of Panel Characteristics on Ethnic Food Perception

In this study, we observed cross-cultural differences in the ratings of familiarity, ethnic authenticity, and liking. It is noteworthy that the SA panel accepted the samples having K-style components better compared to the K panel’s liking of the samples having SA-style components. Moreover, while we initially assumed that incorporation of SA-style components would increase the SA panel’s familiarity scores, the results indicate that K ethnic authenticity and familiarity perceived by the SA panel both decreased when SA ingredients were used in the rice dish samples. This seems to be associated with the SA panel’s low neophobic tendencies, together with frequent exposure to Korean foods. This finding indicates the effect of personal factors, including individual knowledge of food with respect to the perception of ethnic authenticity [48]. Based on the collative-motivation model, flavor liking is driven not only by intrinsic sensory properties but also by a concomitant need for familiarity, novelty, and complexity [49]. Perception of these collative properties was influenced by the level of previous exposure and knowledge [50,51]. Our results suggest that the SA-panel members’ experience with Korean foods largely influenced their interpretation of new samples, thereby reducing the perception of novelty and increasing the familiarity of foreign foods. In addition, their knowledge obtained via exposure to Korean food reduces the cognitive effort required to address new stimuli [52], resulting in a more positive assessment of the sensory properties of the samples.

It has been previously suggested that perceived familiarity and ethnic authenticity are contradictory concepts [53,54]. Contrarily, another study observed empirically that there was an imperfect correlation between novelty and familiarity [55]. Giacalone et al. note that familiarity and novelty lie in slightly different perceptual dimensions and must be evaluated separately [49]. In the present study, the correlation coefficients between the dependent variables (Table 5) supported this argument. The members of the SA panel had been exposed to Korean foods and thus perceived the bibimbap-based samples containing SA components as less familiar as they were capable of judging the samples based on the representations constructed from their own experiences with Korean food [32]. In view of the fact that K ethnic authenticity did not compromise the SA panel’s perception of familiarity, the results suggest that ethnic authenticity and familiarity are separate concepts for the SA panel, with an independent effect on the hedonic ratings. This result revealed that the relationship between ethnic authenticity and familiarity varied depending on the panel’s specific experiences and knowledge.

### 4.3. Limitations

The current study had several limitations offering avenues for future research. The primary limitation relates to the subjects. A number of participants in the SA panel had lived in Korea for >1 year and had significant experiences with Korean food (Table 2). Deliza and MacFie have noted that the level of prior experience with an ethnic food has an important effect on liking [43]; this limits the generalization of the findings beyond the subject groups that participated in this study. The current study was not designed to examine the moderating effects of previous experiences on liking and perception of an ethnic food because the study participants were homogeneous with respect to a high exposure level. As such, it will be necessary to conduct a similar research trial on participants that include overseas consumers with less exposure to Korean food.

Another limitation is that our experimental setting was not ecological, conducted in a controlled lab setting that does not represent real consumption situation. For example, expected liking and appearance liking were assessed only visually using the whole-portioned sample, whereas other response variables were evaluated by tasting a small-sized portion of sample. These factors might have interrupted the holistic sensory experience related to the entire eating process as the study design resulted in a separation between the visual and other sensory experiences. For example, in this study, plating only influenced the expected and appearance liking. This result implies that the effect of visual cues is limited to the formation of expectations and perceptions of experience, but we cannot rule out the possibility that the unrealistic setting might be a confounding factor.

Hedonic responses depend on visual images [56]; as such, it is clear that appearance would contribute to the flavor and texture liking assessed in tasting session. A more ecological experimental setting and design might uncover the role of plating in hedonic responses and perception.

Finally, we did not explore the effect of flavor modification. Although adjustment of the ingredients and/or the cooking process contributes to changes in flavor to some degree, the size and impact of this effect may be much smaller than direct flavor modification achieved by replacing a condiment or a spice with those from another culture. Flavor modification was not considered in this study because previous studies reported adjustment of flavor modification was not successful in increasing foreign consumer’s liking for ethnic foods, and it was assumed that the effect of flavor modification would dominate perception of ethnic authenticity and familiarity based on the theory of flavor principles [15]. However, it would be still worth investigating the interactions between flavor and the components investigated in this study and also their effect on liking and perception of ethnic authenticity and familiarity.

### 4.4. Practical Implications

Localization of an ethnic food by modifying ingredients or the cooking process may present the risk of becoming “mixed”; this creates a confusing overlap between food identities if consumers’ reactions are not fully taken into account [57]. It would be important to have a significant understanding of the target consumers’ needs for ethnic foods, including complexities regarding the correct balance of security obtained from familiarity and the desire for excitement and exotic experience presented by ethnic authenticity. The findings in the present study have practical implications in that they provide information on how specific modifications influence consumer responses; as such, they can be used to explore potential strategies directed toward improving liking for ethnic foods. One of the insights from this study is the effect of using local ingredients. Our results suggest that modification of the ingredients had the largest effect on liking, familiarity, and ethnic authenticity. In the present study, the SA cooking process of stir-frying increased the overall sensory liking due to the enhancement in flavor. It was reported that the aroma of compounds, such as methyl dodecanoate, hexyl octanoate, and oleic acid, which are generated during the frying process, enhances the fatty odor and sweet odor of the fried rice [58]. Although plating exhibited only a limited influence on consumers’ liking and perception, it should not be neglected. The results of one study suggested that “balancing” on a plate could affect the liking of a given food [59]. Overall, the samples representing K plating have multicolored balanced food properties that typically score higher in visual attractiveness than single-colored or imbalanced samples [30]. Additionally, as discussed above, in real-life consumption situations where visual information from plating can be fully integrated with the tasting experience, plating may have a greater influence on the liking and perception of ethnic authenticity.

### 4.5. Conclusions

Consumer perception of ethnic authenticity was affected by the components, mainly the ingredients and the cooking process. Plating had only limited influence on the overall outcomes, notably with respect to the expected liking and appearance liking. Ethnic congruence of the components reduced the cognitive dissonance and ultimately resulted in increased liking. The overall effect of the modification of ingredients, plating, and cooking process was influenced by previous exposure to foreign foods. Consumers who have been exposed to ethnic foods perceived the foods containing foreign components as more familiar and thus more acceptable. Moreover, the level of exposure to a foreign food seems to be associated with how a consumer develops the concept of familiarity and ethnic authenticity. Those who had experienced foreign ethnic foods tend to consider these two concepts within separate dimensions, and not diametrically opposed to one another. Outcomes from the present study have practical implications as they can provide researchers and product developers with guidelines with respect to the extent to which components of an ethnic food can be modified to increase familiarity and liking without an undesirable loss of ethnic authenticity.

## Figures and Tables

**Table 1:** Eight formulations of rice dish samples prepared with a 2^3^ factorial combination of plating, cooking process, and ingredient in Korean or Southeast Asian styles.

	Plating	Korean (Black Stone Bowl, Toasted Sesame)		Southeast Asian (Banana Leaf, Cucumber Garnish)	
	Cooking Process	Korean (Mixing)	Southeast Asian (Stir-Frying)	Korean (Mixing)	Southeast Asian (Stir-Frying)
Ingredients	Korean (spinach, julienned carrot, bean sprout, stir-fried beef, cooked white rice)	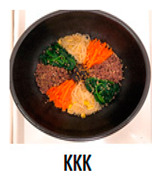	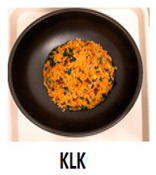	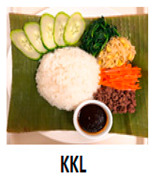	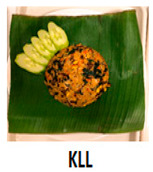
	Southeast Asian (spring onion, chopped carrot, onion, stir-fried beef, cooked Jasmine rice)	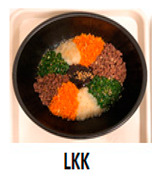	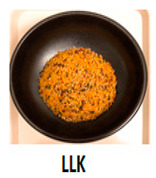	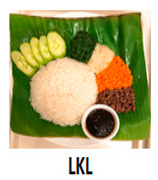	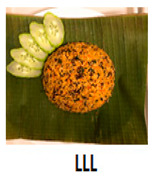

**Table 2:** Participants’ demographics and ethnic foods consumption habits.

Southeast Asian Participants	Percent (%)	Korean Participants	Percent (%)
Classification		Classification	
Gender		Gender	
Male	29.9%	Male	45.8
Female	70.1%	Female	54.2
Nationality			
Indonesia	32.5		
Malaysia	67.5		
Age		Age	
18**–**19	15.6	18**–**19	1.4
20**–**29	79.2	20**–**29	86.1
30**–**39	5.2	30**–**39	12.5
Previous experience in consumption of Korean foods		Previous experience in consumption of Southeast Asian foods	
<6 months	6.5	Yes	93.1
6 month**–**1 year	10.4	No	6.9
1**–**2 years	28.6		
2**–**3 years	14.3	Nationality of Southeast Asian foods that had been tasted before ^1^	
>3 years	40.3		
Duration of Stay in Korea		Vietnam	83.3
<6 months	11.7	Thailand	62.5
6 month**–**1 year	9.1	Malaysia	13.9
1**–**2 years	31.2	Singapore	12.5
2**–**3 years	15.6	Indonesia	8.3
>3 years	32.5	Cambodia	6.9
Consumption frequency of Korean		Experience in eating rice dishes that is same as or similar to southeastern Asian foods tested in this experiment	
foods			
monthly	2.6		
2**–**3 times/month	6.5	Yes	68.2
weekly	15.6	No	31.8
2**–**3 times/week	32.5		
Daily	42.9		
Consumption frequency of bibimbap		Consumption frequency of bibimbap	
<monthly	48.1	<monthly	48.6
2**–**3 times/month	33.8	2**–**3 times/month	41.7
1**–**2 times/week	16.9	1**–**2 times/week	9.7
2**–**3 times/week	1.3	3**–**4 times/week	0.0
Food Neophobic Score	54.7 ± 10.6 ^2^	Food Neophobic Score	51.0 ± 10.2
Variety Seeking Score	5.30 ± 1.07	Variety Seeking Score	5.03 ± 1.01

^1^ Responses were obtained from those who answered to “Yes” to the question asking for previous experience in the consumption of Southeastern Asian foods. ^2^ Mean ± SD.

**Table 3:** F-values associated with the effect of main factors and their 2-way and 3-way interactions on liking ratings, degree of familiarity, perceived Korean (K) and Southeast Asian (SA) ethnicity, and purchase intent.

	Expected Liking	Appearance Liking	Overall Liking	Flavor Liking	Texture Liking	amiliarity	K ethnic Authenticity	SA ethnic Authenticity	Purchase Intent
	*F-Value*								
Nationality	38.06 ***	75.50 ***	20.71 ***	7.75 **	50.44 ***	2.95	16.40 ***	40.16 ***	65.16 ***
Ingredient	31.35 ***	22.81 ***	121.98 ***	103.44 ***	145.50 ***	280.31 ***	1178.73 ***	519.54 ***	133.49 ***
Cooking process	4.65 *	51.911 ***	44.68 ***	39.18 ***	23.60 ***	3.03	7.05 **	13.15 ***	43.96 ***
Plating	3.38	21.16 ***	0.18	0.01	0.06	0.55	6.02 *	3.04	0.69
Nationality × Ingredient	8.98 **	4.40 *	1.93	0.67	15.52 ***	130.89 ***	45.45 ***	191.65 ***	0.00
Nationality × Cooking process	2.18	0.29	0.17	0.19	0.01	0.12	8.95 **	17.80 ***	0.09
Nationality × Plating	6.35 *	1.79	0.23	3.43	0.72	0.42	6.02 *	0.10	2.76
Ingredient × Cooking process	14.26 ***	11.92 **	0.65	0.93	1.64	2.51	10.99 **	18.63 ***	0.21
Ingredient × Plating	4.03 *	1.57	2.87	2.05	0.01	0.59	0.26	1.28	0.05
Cooking process × plating	88.22 ***	132.95 ***	3.70	0.26	2.12	0.03	1.15	1.32	9.77 *
Nationality × Ingredient × Cooking process	0.90	2.73	1.90	2.59	0.00	0.14	1.37	0.56	0.00
Nationality × Ingredient × Plating	0.29	0.39	0.13	2.51	0.20	0.01	2.30	2.21	0.51
Nationality × Cooking process × Plating	6.814 *	4.64 *	1.02	0.13	1.86	0.20	1.99	0.83	0.09
Ingredient × Cooking process × Plating	1.371	1.17	0.13	0.30	0.71	1.82	3.14	0.52	0.21

*** *p* < 0.001, ** *p* < 0.01, * *p* < 0.05.

**Table 4:** Mean scores and *p*-values of hedonic ratings, familiarity, ethnic authenticity, and purchase intent according to the main factors that had significant effects.

	Nationality			Ingredient			Cooking Process			Plating		
	K	SA	*p*-Value ^1^	K	SA	*p*-Value	K	SA	*p*-Value	K	SA	*p*-Value
Expected liking	6.0 ^2^ (1.5)	6.3 (1.7)	<0.001	6.3 (1.6)	6.0 (1.7)	0.001	6.2 (1.7)	6.1 (1.6)	n.s. ^3^	6.2 (1.6)	6.1 (1.6)	N.S. ^3^
Appearance liking	5.8 (1.8)	6.3 (1.8)	<0.001	6.2 (1.8)	5.9 (1.9)	0.003	6.3 (1.9)	5.8 (1.7)	<0.001	6.2 (1.8)	5.9 (1.8)	0.004
overall liking	6.3 (1.5)	6.6 (1.6)	0.003	6.8 (1.5)	6.1 (1.6)	<0.001	6.2 (1.6)	6.7 (1.6)	<0.001	6.4 (1.6)	6.5 (1.6)	N.S.
flavor liking	6.4 (1.6)	6.5 (1.7)	n.s.	6.8 (1.6)	6.1 (1.7)	<0.001	6.3 (1.7)	6.7 (1.7)	<0.001	6.5 (1.7)	6.5 (1.7)	N.S.
texture liking	6.0 (1.8)	6.6 (1.7)	<0.001	6.8 (1.6)	5.9 (1.8)	<0.001	6.1 (1.8)	6.5 (1.7)	<0.001	6.3 (1.8)	6.3 (1.8)	N.S.
familiarity	6.2 (2.0)	6.4 (1.9)	N.S.	6.9 (1.9)	5.7 (1.8)	<0.001	6.2 (2.1)	6.4 (1.8)	N.S.	6.3 (2.0)	6.3 (2.0)	N.S.
K ethnic authenticity	4.2 (1.9)	4.4 (1.9)	0.024	5.4 (1.6)	3.3 (1.6)	<0.001	4.4 (2.0)	4.2 (1.9)	n.s.	4.4 (1.9)	4.2 (1.9)	n.s.
SA ethnic authenticity	3.4 (1.7)	3.7 (1.8)	<0.001	2.9 (1.6)	4.2 (1.6)	<0.001	3.5 (1.7)	3.7 (1.7)	0.030	3.5 (1.8)	3.6 (1.7)	N.S.
Purchase intent	4.3 (1.6)	4.8 (1.7)	<0.001	4.9 (1.6)	4.2 (1.6)	<0.001	4.3 (1.7)	4.8 (1.6)	<0.001	4.6 (1.6)	4.5 (1.7)	N.S.

^1^*p*-values associated with an independent *t*-test conducted as a post-hoc analysis of ANOVA. ^2^ Mean scores (standard deviation). ^3^ Capital N.S. indicates no significant effect of main factors tested using an ANOVA. Small n.s. indicates no significant difference between two treatments in an independent *t*-test conducted as a post-hoc analysis of ANOVA.

**Table 5:** Correlation coefficients between dependent variables by the panels.

	K Panel					SA Panel				
	K Ethnic Authenticity	SA Ethnic Authenticity	Familiarity	Overall Liking	Purchase Intent	K Ethnic Authenticity	SA Ethnic Authenticity	Familiarity	Overall Liking	Purchase Intent
K ethnic authenticity	1					1				
SA ethnic authenticity	−0.751 **	1				−0.286 **	1			
Familiarity	0.824 **	−0.674 **	1			0.150 **	0.477 **	1		
Overall liking	0.373 **	−0.217 **	0.484 **	1		0.175 **	0.416 **	0.552 **	1	
Purchase Intent	0.310 **	−0.130 **	0.397 **	0.801 **	1	0.179 **	0.412 **	0.545 **	0.800 **	1

** The correlation is significant at 0.01.

## Appendix A

**Table A1:** Preparation Procedure of the Samples.

Ingredients	Cooking Process	
	Korean Style	Southeast Asian Style
Korean Bibimbap sauce 25 g Microwave-ready instant white rice (Japonica) 300 g Stir-fried beef (ground) 40 g Stir-fried carrot (julienned) 40 g Seasoned bean sprout 40 g Seasoned spinach 40 g Toasted sesame 5 g	1. Microwave instant white rice for 3 min. 2. Mix all ingredients together first with a spoon for 2 min, and with a hand for 1 min. 3. Serve 30 ± 1 g in a disposable bowl. 4. Plating for appearance evaluation Korean style: place 300 g of heated white rice in the black stoneware bowl. Align all the other ingredients in a circle on the cooked rice so that similar colors do not lie side by side. Place the sauce on the center of the circle. Garnish with 5g toasted sesame. Southeast Asian style: Place a piece of banana leaf on a white porcelain plate. Place 300 g of heated white rice on the leaf and the other ingredients around the rice. Serve the sauce in a transparent disposable bowl. Garnish with five pieces of cucumber slices.	1. Microwave instant white rice for 3 min. 2. Put 5 g of cooking oil in the pan and heat for 30 s. Add all ingredients in the pan and stir-fry for 2 min at medium heat 3. Serve 30 ± 1 g in a disposable bowl. 4. Plating for appearance evaluationKorean style: place 300 g of fried rice in the black stoneware bowl. Garnish with 5 g toasted sesame. Southeast Asian style: Place a piece of banana leaf on a white porcelain plate. Place 300 g of fried rice on the leaf. Garnish with five pieces of cucumber slices.
Southeast Asian Bibimbap sauce 25 g Cooked Jasmine rice 300 g Stir-fried beef (ground) 40 g Stir-fried onion (size) 40 g Stir-fried carrot (size) 40 g Stir-fried scallion (size) 40 g	1. Cook Jasmine rice 2. Mix all ingredients together first with a spoon for 2 min, and with a hand for 1 min. 3. Serve 30 ± 1 g in a disposable bowl. 4. Plating for appearance evaluation Korean style: place 300 g of cooked Jasmine rice in the black stoneware bowl. Align all the other ingredients in a circle on the cooked rice so that similar colors do not lie side by side. Place the sauce on the center of the circle. Garnish with 5 g toasted sesame. Southeast Asian style: Place a piece of banana leaf on a white porcelain plate. Place 300 g of cooked Jasmine rice on the leaf and the other ingredients around the rice. Serve the sauce in a transparent disposable bowl. Garnish with five pieces of cucumber slices.	1. Cook Jasmine rice 2. Put 5 g of cooking oil in the pan and heat for 30 s. Add all ingredients in the pan and stir-fry for 2 min at medium heat 3. Serve 30 ± 1 g in a disposable bowl. 4. Plating for appearance evaluation Korean style: place 300 g of fried rice in the black stoneware bowl. Garnish with 5 g toasted sesame. Southeast Asian style: Place a piece of banana leaf on a white porcelain plate. Place 300 g of fried rice on the leaf. Garnish with five pieces of cucumber slices.
